# PREVENT: Assessing and Improving Knowledge of the Sodium Valproate Pregnancy Prevention Programme in Psychiatric Prescribing

**DOI:** 10.1192/bjo.2022.85

**Published:** 2022-06-20

**Authors:** Laura Havens

**Affiliations:** NHS Greater Glasgow and Clyde, Glasgow, United Kingdom

## Abstract

**Aims:**

The sodium valproate PREVENT programme was introduced by the Medicines and Healthcare products Regulatory Agency (MHRA) in March 2018, and is now a legal requirement due to valproate's risks in pregnancy. This project had two main aims: 1) To assess clinician knowledge of PREVENT, and identify deficiencies in current education. 2) To assess the barriers psychiatrists face in achieving compliance with PREVENT.

**Methods:**

Knowledge and awareness of PREVENT was assessed through an online survey sent to local consultants and specialty doctors in February 2021. The survey included ten questions, four were Likert style to assess attitudes, two assessed local arrangements and four were knowledge based. A free-text section allowed respondents to describe challenges faced implementing PREVENT. Results were analysed, an educational presentation given at local teaching and a poster was created and distributed - both targeting areas of weakest knowledge. A repeat survey was sent out in June 2021, and results collected to reassess.

**Results:**

The pre-teaching survey received twelve responses, the post teaching survey received eleven. In both, 75% of respondents represented general adult services, and 25% represented intellectual disability services.

There was an improvement in confidence of knowledge with all respondents being either “somewhat” (55%) or “very confident” (45%) post-teaching compared to 75% being “somewhat” confident, 10% “unsure” and 17% “very confident” prior to intervention. Pre-teaching, 10% of respondents were unaware that a risk acknowledgement form must be signed annually, while post-teaching 100% correctly identified this should be annual.

Respondents correctly identifying “highly effective” forms of contraception rose from 83% to 100% following teaching. Post-teaching there was an increase of 31% in the number of respondents correctly identifying the necessary documentation where a patient declines the PREVENT programme.

Pre-teaching, half of respondents were unsure if their team had a reminder system for risk acknowledgement forms, and 42% reported having no system. Post-teaching, 27% of respondents reported now having a reminder system in place, and 27% had plans to implement one.

**Conclusion:**

Initial results showed variable knowledge of the PREVENT programme, and a lack of awareness of the administrative requirements including risk acknowledgement forms. Results demonstrated an improvement in knowledge and organisation to help support compliance with the PREVENT programme. Respondents highlighted that knowledge of the PREVENT programme quickly deteriorates given how rarely it is used. Further work includes a full audit of compliance with PREVENT across the health board, as well as considering “refresher” sessions to prevent atrophy of knowledge.

